# Perceived physical exertion during healthcare work and prognosis for recovery from long-term pain in different body regions: Prospective cohort study

**DOI:** 10.1186/1471-2474-13-253

**Published:** 2012-12-19

**Authors:** Lars L Andersen, Thomas Clausen, Roger Persson, Andreas Holtermann

**Affiliations:** 1 National Research Centre for the Working Environment, Lersø Parkalle 105, DK, 2100, Copenhagen Ø, Denmark

**Keywords:** Chronic pain, Musculoskeletal disorders, Neck pain, Back pain, Knee pain, Risk factors, Longitudinal, Eldercare

## Abstract

**Background:**

The prevalence of musculoskeletal pain is high among healthcare workers. Knowledge about risk factors at work is needed to efficiently target preventive strategies. This study estimates the prognosis for recovery from long-term musculoskeletal pain in different body regions among healthcare workers with different levels of perceived physical exertion during healthcare work.

**Methods:**

Prospective cohort study among 4,977 Danish female healthcare workers responding to a baseline and follow-up questionnaire in 2005 and 2006, respectively. We defined long-term pain, short-term pain and pain-free as > 30, 1–30 and 0 days with pain during the last year, and included in the analyses only those with long-term pain at baseline in the low back (N=1,089), neck/shoulder (N=1,400) and knees (N = 579), respectively. Using cumulative logistic regression analysis, the prognosis for recovering from long-term pain at baseline to short-term pain or pain-free at follow-up in the respective body regions when experiencing moderate or light (reference: strenuous) physical exertion during healthcare work was modeled.

**Results:**

Among those with long-term pain at baseline 34% (low back), 29% (neck/shoulders), and 29% (knees) recovered to short-term pain at follow-up and 7% (low back), 8% (neck/shoulders), and 17% (knees) recovered to being pain-free. After adjusting for potential confounders (age, BMI, tenure, smoking status, leisure physical activity and psychosocial work conditions), light perceived physical exertion during healthcare work was associated with improved prognosis for recovery from long-term pain in the low back (OR 1.42, 95% CI 1.01 – 1.99) and neck/shoulders (OR 1.89, 95% CI 1.43 – 2.50), but not in the knees. Moderate physical exertion was not associated with improved prognosis for recovery from long-term pain for any of the body regions.

**Conclusion:**

In the present study, healthcare workers with light perceived physical exertion during healthcare work had the best prognosis for recovery from long-term pain in the low back and neck/shoulders. This suggests that efforts to reduce perceived exertion during work may improve recovery from chronic pain.

## Background

Musculoskeletal pain cause individual distress and great societal costs in terms of poor work ability and sickness absenteeism [1-9]. An increased prevalence of musculoskeletal pain is reported in occupations with high physical work demands [10]. Healthcare work is particularly physically demanding [11] and is often performed by women with low physical capacity [12]. Among more than 8000 healthcare workers, 23%, 28%, and 12% experienced long-term pain in the low back, neck/shoulders, and knees, respectively, which increased the risk for long-term sickness absenteeism by 47-92% [13]. Because many European countries as well as the United States are in shortage of healthcare workers [14,15], finding ways to reduce the prevalence of long-term musculoskeletal pain among this occupational group has enormous relevance.

Biomechanical studies among healthcare workers show high loadings of the low back during patient handling [16,17]. Basic physiological principles dictate adequate rest after strenuous physical exertion for optimal recovery [18]. However, recovery from long-term musculoskeletal pain may be difficult when exposed to daily strenuous healthcare work. Further, the prognosis for recovery from pain is worse among women than men [19]. The highly dynamic pattern of musculoskeletal pain leads to natural recovery among some workers, while others will suffer recurrent episodes or develop more persistent pain [20]. Sparse knowledge exists on how work-related factors influence this differential course of pain recovery. Thus, determination of prognostic factors for recovery from long-term pain among female healthcare workers seems highly relevant.

Perceived physical exertion relates closely to measured work demands relatively to the individual physical capacity - both in terms of cardiovascular [21] and muscular loadings [22]. Thus, perceived physical exertion provides information on the balance between work demands and the capacity to perform the work. Perceived physical exertion can therefore be modulated both by lowering physical work demands (e.g. using assistive devices) and by increasing the individual physical capacity (e.g. through physical conditioning programs). Cross-sectional studies have shown positive associations between perceived physical exertion and low back pain among healthcare workers [23]. However, no studies have investigated the association between perceived physical exertion during healthcare work and the prognosis for recovery from long-term musculoskeletal pain in different body regions. Because perceived physical exertion is easy to assess at the workplace a great potential may exist in using this parameter in regulatory efforts to reduce long-term pain.

The aim of our prospective cohort study was to estimate the prognosis for recovery from long-term pain in different body regions among healthcare workers with different levels of perceived physical exertion during healthcare work. The study population consisted of subgroups with long-term pain in the low back, neck/shoulder, and knees, respectively, selected from a larger cohort of healthcare workers.

## Methods

### Study design and population

This prospective cohort study involves healthcare workers in eldercare that were recruited from 36 municipalities in Denmark. The municipalities are the main providers of eldercare services in Denmark. Accordingly, the municipalities were a necessary partner in the completion of the cohort study as the municipalities provided the research team with the contact details of the study population. The baseline survey started in the winter of 2004, but the main data collection took place in the spring of 2005. Data collection for follow-up was, for the most part, conducted in the autumn of 2006, but continued on to spring 2007. At baseline, questionnaires went out to 12,744 workers from the healthcare sector. Of these potential respondents, 9,949 (78%) completed the questionnaire. One municipality decided to withdraw from the cohort during the follow-up period due to lack of employee-support for taking part in the follow-up study, which meant that the follow-up population consisted of 9,847 employees from 327 individual workplaces in the eldercare services. Of these, 7,864 were eligible for the follow-up questionnaire in 2006, of which 6,307 responded (80%). Further, to increase the focus of the study we excluded male respondents (N=234) and respondents who were not directly engaged in the provision of care services (N=1,021). Thus, the group of healthcare workers with both baseline and follow-up questionnaire replies consisted of 5,052 women being directly engaged in the provision of health-related care services in the Danish eldercare-sector. Due to missing questionnaire replies the data set consisted of 4,977 complete responses regarding the questions about physical exertion and musculoskeletal pain. Because of the method of data collection, survey responses were not obtained from employees who had left the company at follow-up.

### Ethical approval

The study has been notified to and registered by Datatilsynet (the Danish Data Protection Agency). According to Danish law, questionnaire and register based studies do not need approval by ethical and scientific committees, nor informed consent. All survey responses were held confidential as respondents returned their completed questionnaires directly to the research group and confidentiality was maintained by using numbers to identify participants.

### Definition of subgroups with long-term pain at baseline

At baseline, participants replied to the standardized Nordic questionnaire on musculoskeletal pain symptoms in the low back, neck/shoulders and knees [24]. We subsequently defined three groups of healthcare workers with long-term pain (> 30 days during last year) at baseline, and to whom there was information on perceived exertion at baseline and musculoskeletal pain at both baseline and follow-up: 1) long-term low back pain at baseline (N = 1,089), 2) long-term neck/shoulder pain at baseline (N = 1,400), and 3) long-term knee pain at baseline (N = 579). The three pain-groups were not mutually exclusive.

### Prognostic factor: Perceived physical exertion during healthcare work

Participants replied to the following question based on Borg’s Rate of Perceived Exertion scale (RPE): “How would you rate your physical exertion during working with the patients?”. Participants replied on a 7-point scale: 1 = ‘very, very light’, 2 = ‘very light’, 3 = ‘light’, 4 = ‘moderately strenuous’, 5 = ‘strenuous’, 6 = ‘very strenuous’, 7 = ‘very, very strenuous’ [25]. Subsequently for the main statistical analyses, we collapsed the first three response options to ‘light physical exertion’, the fourth to ‘moderate physical exertion’ and the last three to ‘strenuous physical exertion’.

### Outcome variables

At follow-up, participants again replied to the standardized Nordic questionnaire on musculoskeletal pain symptoms in the low back, neck/shoulders and knees [24]. The questionnaire concerned duration of pain in each respective body region during the last year. For the statistical analyses, we defined long-term pain, short-term pain and pain-free as > 30, 1–30 and 0 days with pain during the last year [13,26].

### Confounders

Potential confounders included age (continuous variable), body mass index (BMI = weight/ height^2^, where BMI<25 is normal weight, BMI 25–30 is overweight, BMI > 30 is obese), smoking status (dichotomous variable depicting smoker/non-smoker), leisure physical activity (4-categories from low to a very high level of leisure physical activity) [13,27] and four indicators of perceived psychosocial work conditions from the Copenhagen Psychosocial Questionnaire (COPSOQ) [28,29]: emotional demands, role conflicts, influence at work, and quality of leadership (normalized on a 0–100 scale according to the test-score manual).

### Statistics

Because the outcome variable was trichotomous (long-term pain, short-term pain, pain-free) we used cumulative (i.e. ordered) logistic regression to estimate the prognosis for recovery from long-term pain at baseline to short-term or pain-free at follow-up in the three respective body regions. Odds ratios (OR) and 95% confidence intervals (95% CI) were calculated for the different types of body regions. In Model 1, we adjusted the analyses for age. In Model 2, we additionally adjusted for BMI, tenure, smoking status, and leisure physical activity. Finally, in Model 3, we additionally adjusted for psychosocial work conditions. Because the participants were clustered in workplaces, observations were not statistically independent [30]. We used PROC GENMOD of SAS version 9.2 for all analyses and used the ‘repeated subject’ option to adjust for random effects at the workplace level.

## Results

Table 1 presents descriptive statistics for the main study variables. Of the 4,977 healthcare workers who replied to both the baseline and follow-up questionnaire 22%, 29%, and 12% had long-term pain at baseline in the low back, neck/shoulders, and knees, respectively. Among those with long-term pain at baseline 34% (low back), 29% (neck/shoulders), and 29% (knees) recovered partially (i.e. had short-term pain at follow-up) and 7% (low back), 8% (neck/shoulders), and 17% (knees) recovered fully (i.e. were pain-free at follow-up). 

**Table 1:** Descriptives of the study population of female healthcare workers (N=4,977), and the three subgroups with long-term pain in the low back (N=1,089), neck/shoulders (N=1,400) and knees (N=579)

	**All (N = 4,977)**		**Low back pain(N = 1,089)**		**Neck/shoulder pain(N = 1,400)**		**Knee pain(N = 579)**	
	**Mean (SD)**	**%**^**a**^	**Mean (SD)**	**%**^**a**^	**Mean (SD)**	**%**^**a**^	**Mean (SD)**	**%**^**a**^
Age (years)	46 (9)		47 (8)		47 (8)		49 (8)	
Tenure (years)	9 (7)		9 (7)		9 (7)		10 (7)	
Weekly working hours (h)	31 (4)		31 (5)		31 (5)		31 (4)	
Smoke		36		39		37		36
Body Mass Index								
Normal		59		53		58		49
Overweight		29		33		30		33
Obese		12		14		12		18
Leisure physical activity								
Low		4		3		3		3
Medium		41		44		43		45
High		51		48		49		47
Very high		4		4		4		4
> 30 days with pain last year								
Low back		22		100		45		49
Neck/shoulder		29		58		100		52
Knees		12		27		22		100
Perceived physical exertion during healthcare work								
Light		34		19		23		27
Moderate		41		41		40		38
Strenuous		25		40		37		35
Psychosocial work conditions (0–100)								
Emotional demands	47 (18)		50 (18)		50 (18)		50 (18)	
Influence at work	46 (20)		42 (20)		42 (20)		44 (20)	
Role conflicts	41 (15)		43 (15)		43 (15)		42 (15)	
Quality of leadership	58 (21)		54 (22)		54 (21)		55 (22)	

^a^ "%" refers to percentage of study population.

Among those who replied only to the baseline questionnaire (e.g. due to leaving the workplace during the follow-up period) 25%, 28%, and 12% reported long-term pain at baseline in the low back, neck/shoulders, and knees, respectively.

Table 2 summarizes the odds ratios from the cumulative logistic regression for recovering from long-term pain in the low back, neck/shoulders, and knees when experiencing light and moderate (reference: strenuous) perceived physical exertion during healthcare work. In Model 1, adjusting for age, light perceived physical exertion during healthcare work improved the prognosis for recovery from long-term low back pain (OR 1.61, 95% CI 1.17-2.21) and long-term neck/shoulder pain (OR 2.02, 95% CI 1.55-2.63). In Model 2, adjusting for age, BMI, tenure, smoking status, and leisure physical activity, these findings remained. In Model 3, with additional adjustment for psychosocial work conditions, light perceived physical exertion still improved the prognosis for recovery from long-term pain in the low back (OR 1.42, 95% CI 1.01 – 1.99) and neck/shoulders (OR 1.89, 95% CI 1.43 – 2.50) at follow-up. Light physical exertion was not associated with improved prognosis for recovery from long-term knee pain. Moderate perceived physical exertion was not associated with improved recovery from long-term pain for any of the body regions.

**Table 2:** Prognosis for recovery from long-term pain (>30 days with pain during last year) in the low back, neck/shoulders, and knees at follow-up

			**Model 1**		**Model 2**		**Model 3**	
**Condition at baseline**	**Perceived exertion**	**N**	**OR**	**95% CI**	**OR**	**95% CI**	**OR**	**95% CI**
Long-term low back pain	Strenuous	434	1.00	-	1.00	-	1.00	-
(N = 1089)	Moderate	448	1.21	0.94 - 1.55	1.14	0.87 - 1.48	1.05	0.80 – 1.37
	Light	207	1.61	1.17 - 2.21	1.57	1.13 - 2.19	1.42	1.01 – 1.99
Long-term neck/ shoulder pain	Strenuous	515	1.00	-	1.00	-	1.00	-
(N = 1400)	Moderate	562	1.21	0.94 - 1.56	1.18	0.90 - 1.53	1.10	0.84 – 1.45
	Light	323	2.02	1.55 - 2.63	1.97	1.50 - 2.60	1.89	1.43 – 2.50
Long-term knee pain	Strenuous	206	1.00	-	1.00	-	1.00	-
(N = 579)	Moderate	221	1.01	0.72 - 1.41	0.98	0.69 - 1.38	0.93	0.65 – 1.34
	Light	152	1.28	0.86 - 1.90	1.22	0.80 - 1.87	1.00	0.62 – 1.63

*) among those without pain in this specific region at baseline.

Model 1: Adjusted for age.

Model 2: Adjusted for age, BMI, smoking, tenure, and leisure physical activity.

Model 3: Adjusted for age, BMI, smoking, tenure, leisure physical activity, and psychosocial work environment.

The odds ratios (OR’s) from light and moderate perceived physical exertion are given, referencing strenuous perceived physical exertion.

On an exploratory basis we also tested Model 3 with additional adjustment for co-existing pain in the other body regions. This decreased the OR’s slightly. Light perceived physical exertion became borderline significant for improving the prognosis for recovery from long-term pain in the low back at follow-up (OR 1.35, 95% CI 0.95 – 1.91), and remained significant for the neck/shoulders (OR 1.74, 95% CI 1.32 – 2.31). Co-existing pain in the neck/shoulders worsened the prognosis for recovery from long-term pain in the low back (OR 0.70, 95% CI 0.58 – 0.85), and co-existing pain in the low back worsened the prognosis for recovery from long-term pain in the neck/shoulders (OR 0.71, 95% CI 0.59 – 0.85).

## Discussion

Our prospective cohort study shows an association between light perceived physical exertion during healthcare work in Danish municipalities and the prognosis for recovery from recalled pain >30 days/year in the low back and neck/shoulders. By contrast, light physical exertion did not improve the prognosis for recovery from long-term knee pain, and moderate physical exertion did not improve the prognosis for recovery from long-term pain for any of the body regions.

Healthcare workers experiencing light physical exertion at work had 42% and 89% higher chance for recovering from long-term pain in the low back and neck/shoulders than those experiencing strenuous physical exertion (Model 3). Because the analyses were adjusted for several potential confounders including age, BMI, tenure, smoking status, leisure physical activity, and four psychosocial work environment factors, the results indicate that light physical exertion is a strong and independent positive prognostic factor for recovery from long-term pain in these body regions.

Patient-handling such as moving, turning, lifting, and repositioning patients involves high biomechanical loadings of the low back [16]. The biomechanical load of the neck/shoulder muscles may also be high during healthcare work. Previous cross-sectional studies have shown positive associations between perceived physical exertion and low back pain among healthcare workers [23,31]. A high-quality prospective cohort study found increased risk for developing low back pain from frequent positioning of patients in bed [32]. However, although much research about physical risk factors in the work environment exists, information on the association between perceived exertion and prognosis for recovery from long-term pain in different body regions among healthcare workers has been lacking. Our study expand on previous findings by showing improved prognosis for recovery from long-term low back pain and neck/shoulder pain when healthcare work is performed at exertion levels experienced as light. By contrast, moderate perceived physical exertion did not improve the prognosis for recovery from long-term pain in any of the three body regions. Theoretically, moderate exertion induces less physical strain and may allow the worker to more fully recover between workdays. Speculatively, this may not be adequate when in long-term pain.

Although we did not investigate the mechanisms of recovery from long-term pain, it is plausible that daily strenuous physical exertion does not allow for optimal musculoskeletal recovery between working days. Higher perceived need for recovery has been associated with both work demands and subjective health complaints in different occupational groups [33]. Drawing on principles from exercise physiology, adequate rest after strenuous physical exertion is vital for optimal recovery [18]. In exercise physiology this is often termed overtraining or overreaching, where full recovery is not achieved for an extended period of time and different symptoms accumulate.

In the present study we performed the analyses with three non-mutually exclusive groups based on the anatomical localization of pain. It can be speculated that co-existing pain in either body region can influence the prognosis for recovery. Thus, on an exploratory basis we tested the final statistical model with adjustments for co-existing pain in the two other body regions. This analysis showed that co-existing pain in the neck/shoulders worsened the prognosis for recovery from long-term pain in the low back, and likewise co-existing pain in the low back worsened the prognosis for recovery from long-term pain in the neck/shoulders. Consequently, co-existing pain in the neck/shoulders and low back decreases the likelihood for recovery in either region.

Previous cross-sectional studies have shown associations between perceived physical exertion and knee pain among healthcare workers [31]. In our prospective cohort study, the level of physical exertion during healthcare work did not significantly influence recovery from long-term knee pain. There may be several explanations for this finding. First, perceived physical exertion during healthcare work may only be weakly related to knee strain, i.e. patients are often handled from a bed or chair with minor need for knee bending. Second, the etiology of knee pain may be quite different from that of back or neck/shoulder pain.

There are both strengths and limitations of our study. The large sample size, the high initial response percentage to the baseline questionnaire, and the prospective design increase the validity of our findings. Also, the inclusion of 36 different municipalities increases the external validity. However, as a consequence of our sampling strategy, which entailed delivering the questionnaires to the participants via the workplace in specific municipalities, only participants who remained employed in the municipalities received the follow-up questionnaire. This procedure excluded circa 20% of the participants who for various reasons (e.g. geographical relocation, poor health, or new interests) stopped working in the eldercare services in the participating municipalities. Nevertheless, because additional analyses showed that the proportion of employees with pain > 30 days during the last year was roughly similar between those who left and those who stayed at the workplace this loss of questionnaire replies is unlikely to introduce substantial bias. Furthermore, including a homogenous group of female healthcare workers reduces the risk for bias from socioeconomic confounding, but at the same time limits the generalisability to females of this occupational group. Future studies should also include other aspects than pain in different body regions, for example, how individual workers cope with the pain. Undeniably, how people perceive and experience and ultimately deal with pain is likely to be an important determinant for whether a person will chose to continue working or leave work due to pain.

## Conclusion

In conclusion, female healthcare workers with light perceived physical exertion during healthcare work have a better prognosis for recovery from long-term pain in the low back and neck/shoulders. This suggests that efforts to reduce perceived exertion during work may improve recovery from chronic pain.

## Competing interests

The authors declare that they have no conflict of interest.

## Authors’ contributions

All authors contributed to conception/design, acquisition of data, or analyses and interpretation of data. LLA drafted the article and all co-authors revised it critically for important intellectual content. All authors approved the final version to be published.

## Pre-publication history

The pre-publication history for this paper can be accessed here:

http://www.biomedcentral.com/1471-2474/13/253/prepub
